# Engaging Communities in Preventing Human Papillomavirus-Related Cancers: Two Boot Camp Translations, Colorado, 2017–2018

**DOI:** 10.5888/pcd17.190250

**Published:** 2020-01-02

**Authors:** Sarah E. Brewer, Anne Nederveld, Matthew Simpson

**Affiliations:** 1 Department of Family Medicine, University of Colorado Anschutz Medical Campus, Aurora, Colorado; 2 Adult and Child Consortium for Health Outcomes Research and Delivery Science (ACCORDS), University of Colorado Anschutz Medical Campus, Aurora, Colorado

## Abstract

Since 2006, a vaccine to prevent human papillomavirus (HPV) infection has been available; however, uptake is suboptimal. To encourage HPV vaccine uptake, we employed Boot Camp Translation (BCT) to develop locally relevant materials in 2 Colorado communities, Mesa County and the Denver metropolitan area (Denver metro). The Mesa County group focused on 2 populations, parents of vaccine-eligible children and young adults. The group identified posters, social media, and educational materials for pediatric primary care settings as venues to deliver their messages. The Denver metro group focused on parents of children with low health literacy. Four messages explain the vaccine and call the selected audience to action. Delivery tactics for that group are social media venues and print education materials, including refrigerator magnets, to remind parents about follow-up dosing. BCT can be adapted to develop locally relevant messages and intervention strategies to address HPV vaccination. Future studies should evaluate the effectiveness of community-derived messages to increase HPV vaccination rates.

**Summary**

**What is already known on this topic?**

Despite data on safety and effectiveness, HPV vaccination remains underused. Boot Camp Translation (BCT) is a process for developing messages to improve local uptake of evidence-based practices.

**What is added by this report?**

BCT was adapted for translation of HPV vaccination evidence to community practice and sample messaging materials are presented.

**What are the implications for public health practice?**

This project demonstrates the potential of BCT for engaging communities in creating and disseminating message interventions. The BCT process results in locally relevant messages that resonate with communities of diverse populations.

## Introduction

Human papillomavirus (HPV) causes several types of cancer, including oral and anogenital malignancies, and cancers attributed to HPV are diagnosed in 42,000 people in the United States annually (1). Vaccination against HPV has been available since 2006 for girls and since 2010 for boys. The current vaccine protects against 9 strains of HPV that are responsible for 90% to 95% of anogenital cancers and 95% of genital warts (2). Predictive models indicate that high levels (80%–100%) of adolescent HPV vaccination can result in near eradication of genital warts and substantial (56%–86%) reductions in anal cancer, cervical cancer, abnormal Papanicolaou test results, and HPV-related genital cancers (3). Evidence indicates that HPV vaccination is safe and effective for preventing HPV infection and has no association with significant adverse effects or early onset of sexual activity (4,5).

Despite data on safety and effectiveness, HPV vaccination is underused. US data for 2017 show that 69% of girls and 63% of boys who were eligible (aged 13–17 years) received the first dose, but only 53% of girls and 44% of boys completed the vaccine series (at least 2 doses, depending on age at first dose) by age 17 years (6). In 2018, 72% of eligible adolescents in Colorado had received 1 dose and 54% completed the series (7).

Given persistent low vaccination rates, lack of knowledge, and attitudinal barriers, engaging people in developing community-specific messages that increase knowledge and encourage HPV vaccination might be more effective than using a general approach to increase vaccine uptake. Community-specific messages have the potential to address local concerns more effectively, be more culturally acceptable to selected audiences, increase risk perception, and begin discussions about vaccination.

## Boot Camp Translation

Boot Camp Translation (BCT) is a community-engaged translational research process for developing community-specific messages to improve local uptake of evidence-based practices. BCT has been used to develop messages for colon cancer screening, mental health, obesity prevention, diabetes, hypertension, and chronic pain (8,9). The process involves a diverse group of 10 to 12 community members (eg, different professions, racial/ethnic groups, or ages) who learn about the scientific evidence on a health topic and strategize together how to translate that evidence into messages for the local community. BCT begins with a kick-off meeting that includes an expert presentation on the topic to increase participant knowledge of current evidence and ease in discussing the topic. After the presentation, participants brainstorm to extract the essential concepts in current evidence and translate them into the following:

- What do we want people to learn?
- Who should learn this?
- How should we communicate this message?

The group creates a draft of key messages at the end of the kick-off meeting. Soon after the kick-off, a series of telephone and in-person meetings are held to refine messages, develop and design messaging materials, and plan dissemination. Each stage in the process results in a set of messaging products and a plan for dissemination within the community.

The goal for our project was to apply the BCT process to develop effective community-responsive messages, materials, and dissemination plans to promote HPV vaccination in 2 Colorado communities. We describe the community engagement process and resulting products of the 2 BCTs in this brief. This is the first use of the BCT process to support HPV vaccination and the first BCTs reported in the literature to engage adolescents.

We conducted BCT in 2 Colorado communities, 1 in urban metropolitan Denver (Denver metro) and 1 in semirural Mesa County in western Colorado. The community of Mesa County is composed of the urban Grand Junction and its surrounding rural communities. Approximately 81% of the Mesa County population is non-Hispanic white (10), and 15% of Mesa County residents live in poverty, compared with 11% statewide. At the start of this project, HPV series completion rates were 21% for girls and 16% for boys in Mesa County (7).

In the Denver metro area, we focused on neighborhoods within the catchment area of a partner agency, 2040 Partners for Health. These neighborhoods are racially, ethnically, linguistically, educationally, and economically diverse. For example, in some of these neighborhoods, 50% of residents live below the federal poverty level (≤$25,100 for a family of 4 in 2017) and less than one-half of adults have a high school diploma (11,12). HPV vaccine series completion rates range from 42% to 53% in the counties where these neighborhoods are located (13).

## Participation

**Figure 1:**
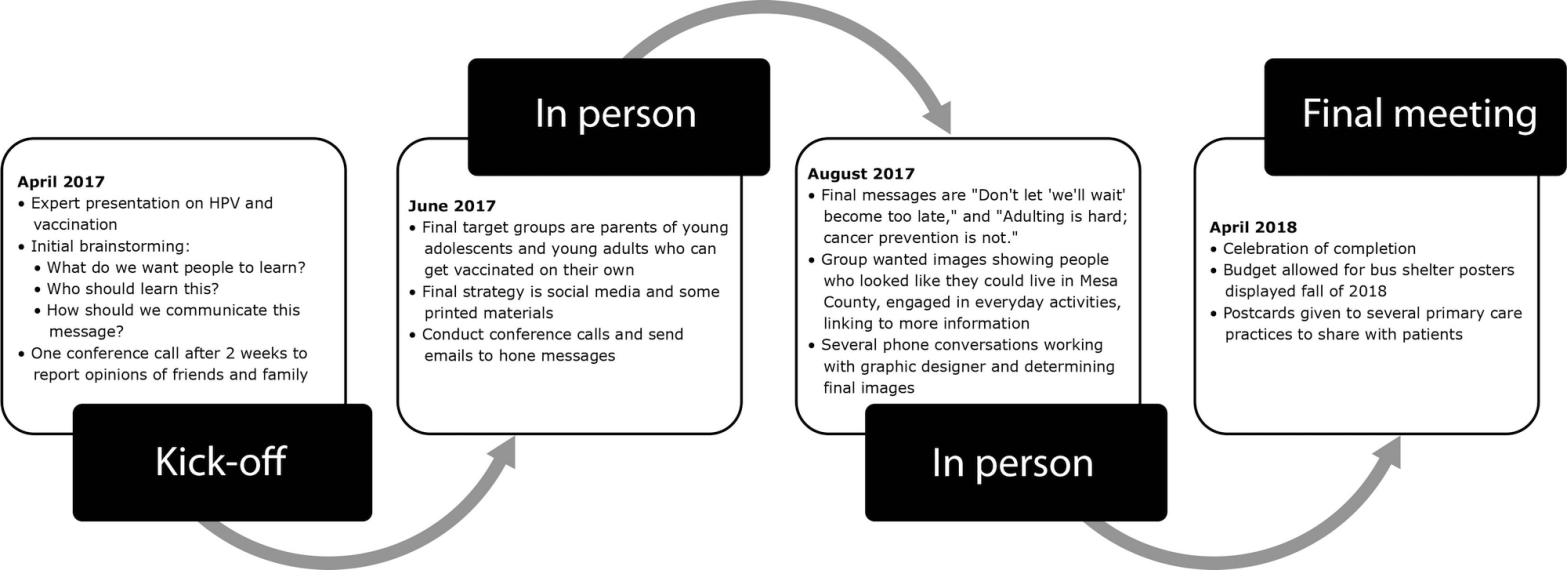
Boot Camp Translation activities timeline in Mesa County, Colorado, 2017–2018. Abbreviation: HPV, human papillomavirus.

Eleven participants represented diverse organizations and backgrounds in the Mesa County BCT. Adolescents, parents, health care providers, public health workers, school district employees and social service providers were included in that BCT. Participants were predominantly female and all under retirement age. Ten people participated in Denver metro BCT, including 2 adolescents, 7 parents, and a diverse representation of human service and public health employees. Participants were from different races/ethnicities, languages, and age groups. The group in Mesa County met in person 4 times, including the kick-off. Five 30-minute conference telephone calls were held. The process lasted about 1 year from spring 2017 to spring 2018, somewhat slower than usual because of delays in product design and development. The Denver metro BCT Group also met in person 4 times, including the kick-off. In addition, the group held 7 half-hour conference calls to discuss aspects of message development. The process lasted 6 months, from fall 2017 to spring 2018 (Figure 1).

## Messages

In Mesa County, participants decided to develop 2 sets of messages, 1 for parents of children aged 9 to 11 years (preteens) and 1 for young adults (aged 18–26 years) who were not yet vaccinated. They chose to focus on parents of preteens after learning that the vaccine is most effective when administered to children in this age group (14). Participants believed that parents wait to vaccinate for HPV because they perceive risk of HPV infection to be low for their children and do not understand the immunologic benefit of vaccinating early. Key messages that the group wanted to convey were

- HPV causes multiple types of cancer, not just cervical cancer.
- The HPV vaccine is recommended for boys and girls.
- The HPV vaccine is more effective when administered to children aged 9 to 14 years than age 15 years or older.

These concepts resulted in the tagline, “Don’t let ‘We’ll wait’ turn into ‘too late.’” The tagline is coupled with images of parents, presumably, and their preteen children of both sexes. Information about effectiveness of the vaccine when given to preteens was also included.

For young adults, participants wanted to capitalize on empowerment and the ability to make one’s own decisions. Resulting key messages were

- It’s not too late to get vaccinated.
- Cancer can happen in young adulthood.
- Self-advocacy and empowerment; vaccination is an adult decision.

**Figure 2:**
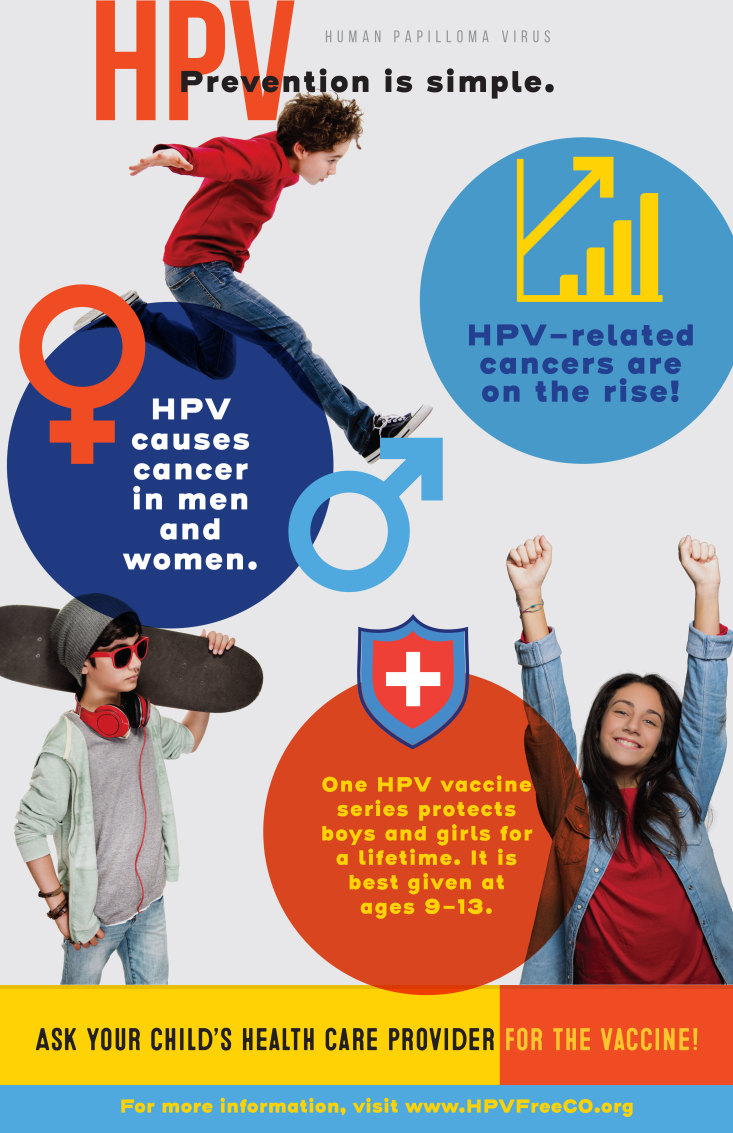
Boot Camp Translation product example for young adults in Mesa County, Colorado.

The group developed the tagline “Adulting is hard; cancer prevention is not,” coupled with images of young adults working, studying, or socializing (Figure 2). Other information in the messages included “HPV causes cancer: cervical, penile, vaginal, oral and anal. Get vaccinated.” Messages for parents of preteens and young adults direct readers to the website www.HPVFreeCO.org.

BCT participants believed social media was an important way to disseminate the messages, particularly for the campaign that focuses on young adults. Images were designed for social media platforms, such as Instagram and Snapchat. This approach would allow for a social media campaign and evaluation of its effectiveness both in messaging and in vaccination rate changes. Postcards, posters, and billboards materials were also developed.

The Denver metro group selected parents of children aged 8 to 13 years with lower health literacy and reduced access to health care and as the target population. The decision to target parents of elementary school children and preteens was motivated by evidence that getting the vaccine before age 15 years is most effective, and by the community’s collective knowledge, that access to primary care, including vaccines, is a challenge in Denver metro neighborhoods, as identified by 2040 Partners for Health. Key messages to convey were

- HPV-related cancers are on the rise!
- HPV causes cancer in men and women.
- One HPV vaccine series protects boys and girls for a lifetime and is best administered at ages 9 to13 years.
- Ask your child’s health care provider for the vaccine!

**Figure 3:**
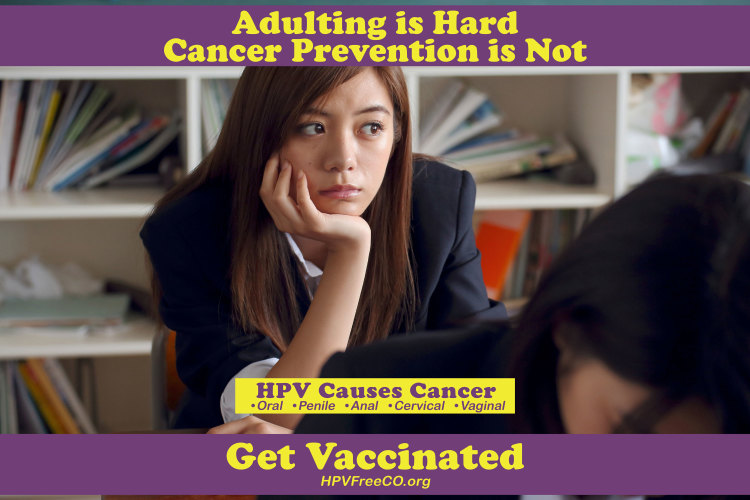
Boot Camp Translation product example for preteens in Metropolitan Denver, Colorado.

The group chose not to develop a tagline; instead, the group integrated messages into an infographic of photographic images and text (Figure 3). The Denver metro campaign also directed readers to the website www.HPVFreeCO.org for more information. However, the group believed it was crucial to provide key information within the messaging, so that it was delivered to readers who did not visit the website.

Flyers, web advertisements, and images that fit social media platforms were developed to ensure messages could reach groups through communication tools that were most accessible to them. Postcards, posters, and billboards were created and shared with BCT group members and distributed by the partner organization. At the time of this publication, both Mesa County and Denver metro groups had produced small-scale print campaign products (posters and postcards) for distribution in local venues. Neither the social media campaigns nor the evaluations have been completed in Mesa County or the Denver metro area.

## Implications for Public Health Practice

This project demonstrates the potential use of BCT as a method for engaging communities in creating and disseminating message interventions and accompanying dissemination plans that are locally relevant and effective. Our BCT processes in 2 Colorado communities show that BCT can 1) be used to encourage HPV vaccination, and 2) result in locally relevant messages that resonate with communities of unique populations.

Reinforcing the idea that public health messages should be locally generated, discussions after the informational presentation differed between the 2 communities. In Mesa County, participants believed that parent-focused messages should emphasize that the vaccine prevents cancer and encourage vaccination of younger children (ages 9–14 years) because of higher vaccine efficacy at this age. Participants believed that vaccine hesitancy in Mesa County partially stemmed from the understanding of HPV as a sexually transmitted infection that young adolescents would not be exposed to yet. Participants noted the importance of focusing on cancer prevention and removing references to sexual activity. They believed that responsibility for vaccination should be encouraged among young adults as a way to protect themselves against cancers from sexually transmitted infections, and messages should emphasize autonomy and the ability to make one’s own health care decisions.

By contrast, in the larger county of Denver, the group determined that the focus should be on misconceptions associated with the HPV vaccine. This group wanted to communicate the long-term prevention effectiveness that the vaccine provides and that a person needs only to receive the vaccine series once. The group decided that parents of elementary school-aged children should hear that the vaccine is best administered early because it is more effective and requires only 2 doses when the series is initiated before age 15 years. This group emphasized 2 barriers to HPV vaccination in their communities. The first barrier is that HPV information for parents is lacking, and the other is that many preteens have insufficient health insurance, resulting in fewer opportunities to see health care providers and get vaccinated. Finally, the Denver metro group wanted to leverage local social connections to distribute the final information and materials from the BCT process. The group agreed that messages would be most effective when delivered by peers in community locations that are frequented by parents, such as recreation centers, churches, food banks, and schools.

Reaching target levels of HPV vaccination rates would be a public health success by greatly reducing morbidity, mortality, and health care costs. Community members are interested in learning more about HPV vaccination and in actively participating in message development for cancer prevention and other important public health topics. Messages developed through processes like BCT, which explores and incorporates community perspectives and voice, can be more accepted and effective than messages developed without community input. The effectiveness of messages developed through community-engaged approaches like BCT should be further explored in the context of vaccination and adolescent health. A small-scale release of the campaign was implemented in both the Denver metro area and in Mesa County. We plan to continue the campaign and evaluate the effectiveness of these locally relevant messages. We also aim to expand the use of the BCT approach to develop messages for HPV vaccination and other adolescent vaccines in more Colorado counties.
